# Unveiling Hypothalamic Molecular Signatures via Retrograde Viral Tracing and Single-Cell Transcriptomics

**DOI:** 10.1038/s41597-023-02789-6

**Published:** 2023-12-04

**Authors:** Muhammad Junaid, Han Kyoung Choe, Kunio Kondoh, Eun Jeong Lee, Su Bin Lim

**Affiliations:** 1 Department of Biochemistry & Molecular Biology, Ajou University School of Medicine, Suwon, 16499 Korea; 2 Department of Brain Sciences, Daegu Gyeongbuk Institute of Science and Technology (DGIST), Daegu, 42988 Korea; 3 Division of Endocrinology and Metabolism, Department of Homeostatic Regulation, National Institute for Physiological Sciences, National Institutes of Natural Sciences, Okazaki, Aichi 444-8585 Japan; 4 Department of Brain Science, Ajou University School of Medicine, Suwon, 16499 Korea

**Keywords:** High-throughput screening, Molecular neuroscience

## Abstract

Despite the importance of hypothalamic neurocircuits in regulating homeostatic and survival-related behaviors, our understanding of the intrinsic molecular identities of neural components involved in these complex multi-synaptic interactions remains limited. In this study, we constructed a Cre recombinase-dependent pseudorabies virus (PRVs) capable of crossing synapses, coupled with transcriptome analysis of single upstream neurons post-infection. By utilizing this retrograde nuclear Connect-seq (nuConnect-seq) approach, we generated a single nuclei RNA-seq (snRNA-seq) dataset of 1,533 cells derived from the hypothalamus of CRH-IRES-Cre (CRH-Cre) mice. To ensure the technical validity of our nuConnect-seq dataset, we employed a label transfer technique against an integrated reference dataset of postnatal mouse hypothalamus comprising 152,524 QC-passed cells. The uniqueness of our approach lies in the integration of diverse datasets for validation, providing a more nuanced diversity of hypothalamic cell types. The presented validated dataset may deepen our understanding of hypothalamic neurocircuits and underscore the essential role of comprehensive integrated transcriptomic data for technical validity.

## Background & Summary

Understanding the complexity of hypothalamic neurocircuits and identifying their molecular signatures is crucial for comprehending the coordinated physiological responses^1–3^. In past years, the high-throughput single-cell RNA sequencing (scRNA-seq) analytical and experimental approaches have facilitated the understanding of the transcriptional cataloging of cell types in the hypothalamus and many other adjacent tissues of the brain^4–6^. However, very little is known about the molecular identification of neural components involved in complex multi-synaptic circuits. In addition, there is a need for a more comprehensive characterization of cell type populations and their distribution to understand the neural circuit properties.

**Fig. 1:**
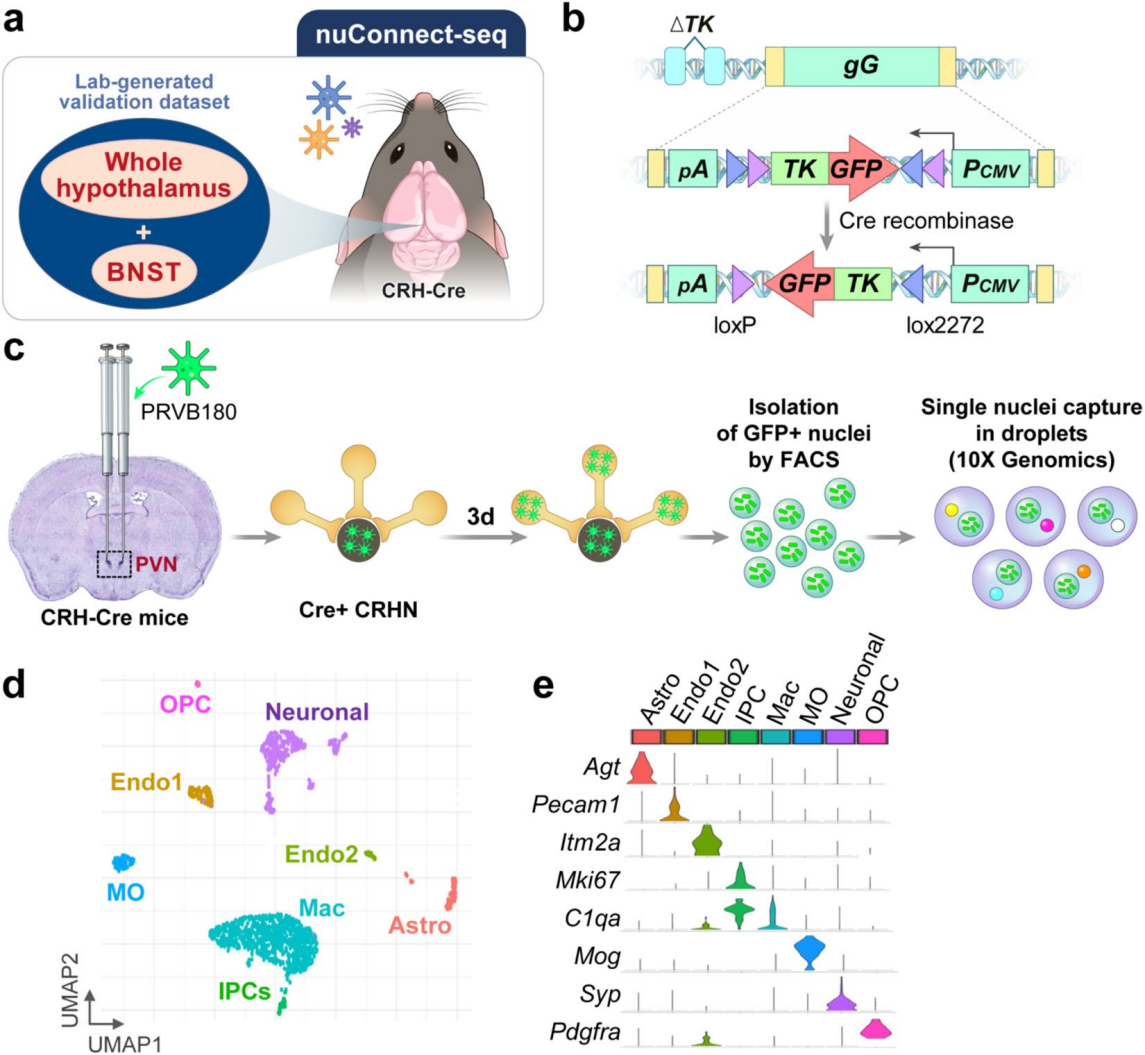
Generation of a nuConnect-seq-derived dataset. (**a**) A retrograde nuConnect-seq approach was used to generate a single nuclei RNA-seq (snRNA-seq) dataset from CRH-IRES-Cre (CRH-Cre) mice. (**b**) PRVB180, a pseudorabies virus expressing TK (thymidine kinase)-GFP (green fluorescence protein) in the Cre+ neurons and their upstream. (**c**) The flow of nuConnect-seq method. (**d**) UMAP plot showing the manually curated cell types of the presented dataset. (**e**) Violin plot showing the relative expression of neuronal and non-neuronal gene markers.

Recent neuroanatomical tracer studies have provided invaluable insights into visualizing and defining complex presynaptic neural circuits^7–9^. Furthermore, these studies have confirmed the faithful retrograde transmission of pseudorabies viruses (PRVs) between interconnected neurons via direct cell-to-cell contact, rather than extracellular diffusion^10–12^. Upon infecting neurons, PRVs can trigger the activation of adjacent astrocytes and microglia, which then function to sequester heavily infected or dying neurons^13,14^. It’s worth noting that glial cells lack the necessary machinery to generate and release infectious (enveloped) virions. The glial cells obtained through Connect-seq likely represent neighboring cells in close proximity to neurons, with specific projections to CRH neurons within the PVN of CRH-Cre mice^8,15^. Here, we focus on a retrograde Connect-seq approach to produce a hypothalamic single nuclei RNA-seq (snRNA-seq) dataset from CRH-IRES-Cre (CRH-Cre) mice. We refer to this approach as “nuclear Connect-seq” (nuConnect-seq), in contrast to the original whole-cell-based Connect-seq method (Fig. 1a–c).

**Fig. 2:**
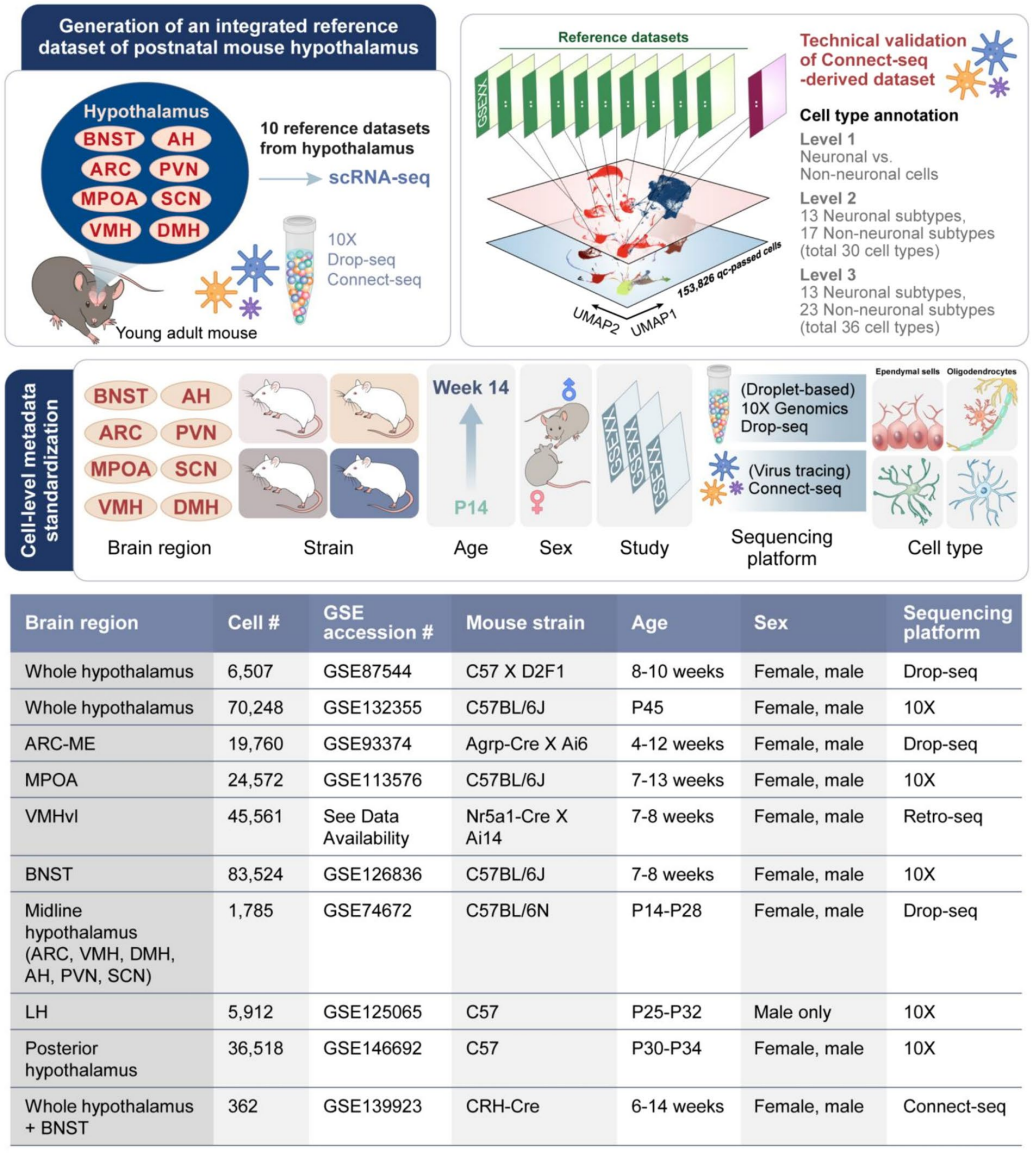
Schematic representation of the generation of an integrated reference dataset of postnatal mouse hypothalamus for technical validation of nuConnect-seq-derived dataset. Ten independent datasets were integrated and batch-corrected in an anchor-based manner. Cell-level metadata was manually curated and standardized for all the datasets. A uniform informatic pipeline was used for clustering and cell type annotation.

In this study, we combine the use of conditional neurotropic viruses that cross synapses with transcriptome analysis of single upstream neurons infected with the virus to identify the molecular identities of upstream CRH neurons in hypothalamic brain regions^8^ (see Methods, Fig. 1). Thus, we created a nuConnect-seq-derived snRNA-seq dataset of 1,533 cells from the hypothalamus and the bed nucleus of CRH-IRES-Cre (CRH-Cre) mice. To further evaluate the technical robustness of our lab-generated nuConnect-seq dataset, we processed and re-analyzed 10 publicly available scRNA-seq transcriptomic datasets, each compromising unique hypothalamic regions of mice. After merging these datasets, we performed an integrative single-cell transcriptomic analysis through anchor-based mapping, comprising 152,524 QC-passed cells with a total of 36 distinct neuronal and non-neuronal cell types (Fig. 2). Then we conducted cell type label transfer between our nuConnect-seq-derived dataset and the integrated transcriptomic reference dataset.

**Fig. 3:**
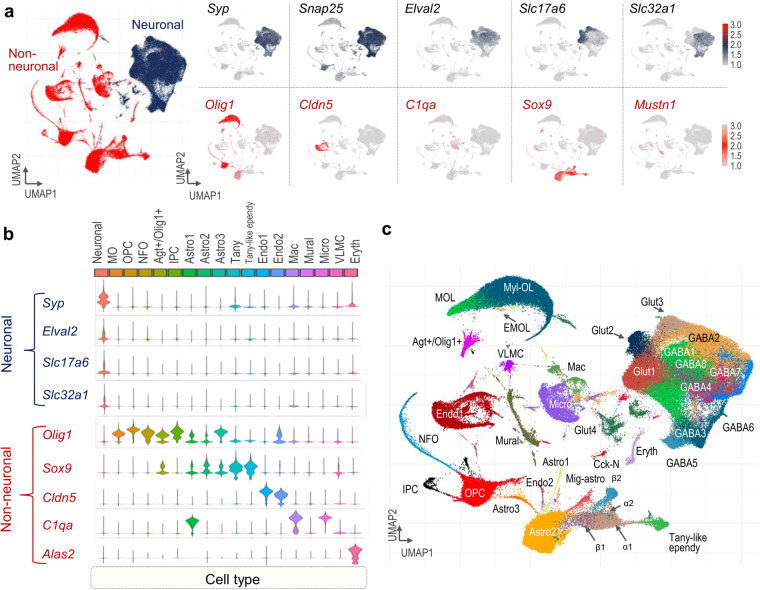
Cell type annotation of the integrated reference dataset of young adult mouse hypothalamus. (**a**) UMAP plots showing neuronal or non-neuronal cells (left) classified by combined expression of pan-neuronal markers (right). (**b**) Relative expression of pan-neuronal and non-neuronal marker genes is shown. (**c**) Final UMAP plot showing subtypes of neuronal and non-neuronal cells.

**Fig. 4:**
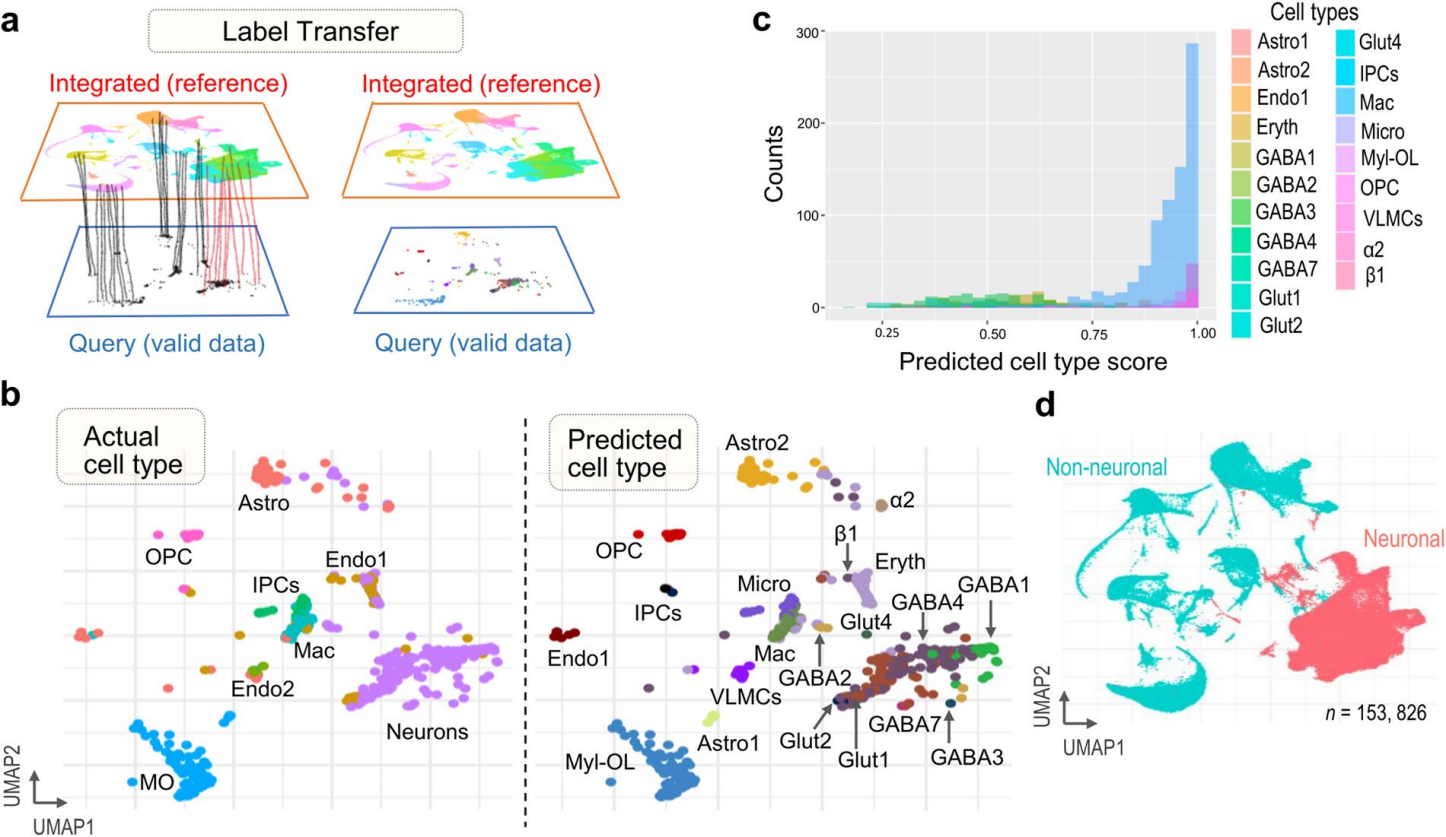
Mapping and transferring labels for technical validation of nuConnect-seq-derived dataset. (**a**) Schematic representation showing identified anchors, which facilitate the transfer of discrete labels between integrated (reference) and lab-generated nuConnect-seq (query) datasets. (**b**) UMAP plots show the actual and predicted cell types on the nuConnect-seq dataset by projecting cells of the nuConnect-seq dataset onto UMAP embeddings. (**c**) Histogram plot showing the distribution of predicted cell type scores across the predicted cell types in the validation dataset. (**d**) UMAP plot showing neuronal and non-neuronal cells of the final merged (integrated and nuConnect-seq dataset).

By following the Seurat label transfer workflow as suggested in the “Mapping and annotating query datasets” vignette, we validated our newly generated nuConnect-sec dataset with the reference map of over 100,000 hypothalamic single nuclei (Figs. 3, 4). Through this approach, we effectively demonstrate the merits of utilizing label transfer to address the inherent limitations posed by the relatively small nuConnect-seq datasets. This comparison against a substantially larger reference dataset highlights the advantages of leveraging label transfer. Finally, our validated nuConnect-seq dataset, along with a large-scale single-cell transcriptomic reference dataset with harmonized cell-level metadata, can serve as an asset for exploring genes across the hypothalamus.

## Methods

### Mice

To produce nuConnect-seq data, CRH-IRES-Cre (CRH-Cre; stock no, 012704) mice were purchased from The Jackson Laboratory. All procedures involving mice were approved by the Ajou University Institutional Animal Care and Use Committee and/or DGIST Institutional Animal Care and Use Committee.

### PRV (pseudorabies virus)

PRVB180 was cultivated following established protocols as detailed in prior studies^8,16^. In brief, PRV180 was propagated by infecting PK15 cells (obtained from the American Type Culture Collection) with the virus, utilizing a multiplicity of infections ranging from 0.1 to 0.01. Following infection, notable cytopathic effects became apparent within approximately two days. The infected cells were subsequently collected through scraping, and the cellular material was swiftly frozen using liquid nitrogen, then rapidly thawed within a water bath at 37 °C. After three freeze-thaw cycles, cellular debris was eliminated through centrifugation, performed twice at 1000 g for 5 minutes each. The resulting supernatant was employed for subsequent experiments. Viral stocks were quantified through standard plaque assays carried out on PK15 cells^17^, with the titer denoted in terms of plaque-forming units (pfu).

### Stereotaxic injections

Viruses were injected into the PVN of CRH-Cre mice as described previously^8^. Viruses were injected into the brain using a Stereotaxic Alignment System (David Kopf Instruments) with inhalation anesthesia of 2% isoflurane. Briefly, 1 µL of PRVs (PRVB180; 1 to 1.5 × 106 p.f.u.) were loaded into a 1-µL syringe and injected bilaterally into the brain at a rate of 100 nL/min. The needle was inserted into the PVN based on a stereotaxic atlas (anterior-posterior (AP), −0.4 mm; medial-lateral (ML), ±0.3 mm; dorsal-ventral (DV), −5.0 mm)^18^. After recovery, animals were singly housed with regular 12-h dark/light cycles, and food and water were provided ad libitum.

### Isolation of single nuclei

nuConnect-seq experiments were performed as previously described^8^ with modifications for nuclei isolations, Adult 8-week-old CRH-Cre male mice were injected with PRVB180. After 3 days, mice were euthanized by cervical dislocation, and the brain was quickly removed. The hypothalamus was carefully micro-dissected under a microscope and immediately placed into a nuclei isolation medium (sucrose 0.25 M, KCl 25 mM, MgCl2 5 mM, Tris-Cl 10 mM, dithiothreitol, 0.1% Triton). Tissue underwent Dounce homogenization, facilitating the mechanical isolation of nuclei from cells^19^. Hoechst 33342 nucleic acid stain (5 μM, Life Technologies) was introduced into the media to enhance nuclei visualization. Subsequently, the samples were washed, reconstituted in a nuclei storage buffer (composed of sucrose, 5 mM MgCl2, and 10 mM TrisCl), and then filtered. It’s important to note that solutions and samples were maintained cold throughout the procedural steps. Then, PRV-infected single nuclei were isolated based on the fluorescence emitted by TK-GFP using flow cytometry (FACSAria II; BD Biosciences) according to methods described previously^8^, with some modifications for isolating single nuclei^20^. Hoechst-positive nuclei were gated first, followed by the exclusion of debris using forward and side scatter pulse area parameters (FSC-A and SSC-A), and the exclusion of aggregates using pulse width (FSC-W and SSC-W). Next, single nuclei with high GFP were placed in ice-cold methanol and then stored at −80 °C immediately. Single-nuclei suspensions obtained from the cortex of CRH-Cre mice injected with PRVB180 were analyzed first in the initial experiments to determine the threshold for fluorescence, and then single-nuclei suspensions from CRH-Cre mice were analyzed to sort GFP+ fluorescent nuclei.

### Single nuclei capture

FACS-sorted single nuclei were loaded onto an independent single channel of a Chromium Controller (10 × Genomics) single-cell platform. In summary, we gathered a cumulative total of 10,000 single nuclei from five CRH-Cre mice, which were subsequently pooled and loaded onto the capture setup utilizing the Chromium Single Cell 3′ Reagent kit with, v2 Chemistry (10 × Genomics). Following the capture process and cellular lysis, complementary DNA was synthesized and amplified through a 14-cycle process following the manufacturer’s instructions (10 × Genomics). The amplified cDNA was then used to create an Illumina sequencing library, which was subsequently sequenced on a single lane of a NovaSeq. 6000 (Illumina). To facilitate FASTQ generation and subsequent alignments, the initial Illumina basecall files (*.bcl) were converted to FASTQs using Cell Ranger v.1.3 (10 × Genomics), which uses bcl2fastq v.2.17.1.14. FASTQ files were then aligned to the mm10 genome and transcriptome using the Cell Ranger v.1.3 pipeline. This pipeline results in the generation of a ‘gene vs cell’ expression matrix.

### nuConnect-seq-derived data

The Connect-seq dataset was processed using the Seurat package v.4.3.0^21^ in R v.4.2.1 (as for integrated reference dataset) (Supplementary Fig. S1). After loading the dataset into R, the Seurat object was created using the “CreateSeuratObject” function. Before main analyses, metadata for each cell was meticulously added, encapsulating various parameters including age (8 weeks), sex (male), region (whole hypothalamus + BNST), technological method (FACS), and mouse strain (CRH-Cre). This cell-level metadata standardization ensures consistency and clarity in our dataset. To mitigate the potential biases arising from disparate sequencing depths across cells, the dataset was normalized using the “LogNormalize” method in Seurat with default parameters. Then the top 2000 variable genes were identified using the FindvariableFeatures() function.

Using these top 2000 variable genes nCount_RNA was regressed out in the scaling step followed by PCA (principal component analysis). Then we employed the UMAP (uniform manifold approximation and projection) on the top 20 principal components in a two-dimensional space to visualize a clear depiction of cell distribution. Further graph-based clustering was performed by using the FindCluster() function in Seurat. For cell clustering, resolution was set as 0.25 to ensure clear granularity and avoid overlap of comparable cells, and 8 cell clusters were identified using specific marker genes, including IPCs (*Mki67*^+^), OPC (*Pdgfra*^+^*)*, Astro (*Agt*^+^), Endo1 (*Pecam*^+^), Endo2 (*Itm2a*^+^), Mac (*C1qa*^+^), MO (*Mog*^+^) and one neuronal cluster expressing *Syp* (Fig. 1).

## Data Records

Raw counts data and processed sequencing data generated in this study have been submitted to the NCBI Gene Expression Omnibus (GEO) under the accession number GSE240765^22^. Our nuConnect-seq dataset is mainly composed of three primary files, (1) “features.tsv.gz”, a tab-separated values (TSV) file that is compressed using gzip, provides a comprehensive catalog of the genomic features considered in the nuConnect-seq dataset. (2) The “barcodes.tsv.gz” file, compressed using gzip, contains the information of unique molecule identifiers that are very important for cell identification. (3) The “matrix.mtx.gz” file represents the sparse matrix where each row represents the genes, and each column represents the individual cell. All these files with the final annotated nuConnect-seq dataset named as “Validation. rds” file containing the information about cell-level metadata, are also deposited in figshare, (10.6084/m9.figshare.21981251.v1)^23^. Furthermore, the matrix data derived from the 10X CellRanger metrics has been provided in Supplementary Table 1.

Our integrated transcriptomic reference dataset named the “final_hypo_ann.rds” is accessible in the figshare repository (10.6084/m9.figshare.21981251.v1)^23^. The data can be used to construct quality control (QC) metrics and obtain standardized cell-level metadata including information on age, brain area, sex, single-cell technology, mouse strain, and manually annotated neuronal and non-neuronal cell types.

## Technical Validation

### Generation of the integrated reference scRNA-seq dataset

To assess the technical validity of our lab-generated nuConnect-seq derived single nuclei RNA-seq (snRNA-seq) dataset, we collected ten publicly available scRNA-seq datasets from the NCBI GEO under the accession numbers GSE87544^24^, GSE132355^25^, GSE93374^26^, GSE113576^5^, GSE126836^27^, GSE74672^28–30^, GSE125065^6^, GSE146692^31^, GSE139923^8^ and 10.17632/ypx3sw2f7c.1 (Mendeley Data)^32^. These datasets of mice originated from various hypothalamic brain regions including arcuate hypothalamus and median eminence (ARC-ME), lateral hypothalamus (LH), medial preoptic area (MPOA), midline hypothalamus, posterior hypothalamus, ventromedial hypothalamus (VMHvl), and the bed nucleus of the stria terminalis (BNST), with ages ranging from P14 to 14 weeks. The datasets were obtained from mice of various strains including C57BL/6, C57 × D2F1, Agrp-Cre × Ai6, Nr5a1-Cre × Ai14, and CRH-Cre^5,6,8,24–27,31,33^, which were further processed using the Seurat package^34^ in R (v. 4.2.1).

To facilitate the re-use of the integrated data, we manually curated cell-level attributes, such as study, age, brain region, sex, and strain of mouse of each dataset, and used standardized texts for metadata (Fig. 2). After merging all these datasets, a threshold of 15% mitochondrial content was chosen based on the observed distribution of mitochondrial percentages across the cells followed by feature counts over 2,800 or less than 100 were filtered out (Supplementary Fig. 2a,b). Cells with very low gene counts also tend to have an abnormally high percentage of mitochondrial genes. But we also were specific regarding the inherent nature of brain cells that specific specialized and quiescent neurons in the brain may have a reduced gene expression profile, leading to inherently lower detection rates. Our threshold ensures that these critical cells are not inadvertently filtered out. Our filtering approach, therefore, not only retains potentially quiescent or low transcript count cells but also effectively removes cells likely to be poor quality or damaged. A total of 152,524-QC passed cells were subsequently included for further analyses, and variable features of these cells were identified after normalization. The FindIntegrateionAnchors() function of the Seurat was applied to merged cells to find the shared cell population across datasets. Then we identified the cross-dataset pairs of cells with similar biological states (“anchors”) to correct for technical differences between datasets. The number of principal components (PCs) of the dataset was determined using the Elbow plot before the clustering of cells (Supplementary Fig. 2c). To comprehensively identify distinct cell populations, cells of the integrated dataset were clustered into distinct cell populations using the FindClusters() function by implementing multiple resolutions (Supplementary Fig. 2d).

Supplementary Fig S3 shows QC-passed cells colored by different cell-level attributes before and after batch removal, further divided by cell type proportion across all datasets. We quantitatively evaluated the quality of clustering results for each resolution to examine whether subpopulations of cells could be split or merged. To ensure clear granularity and avoid overlap of comparable cells in different clusters, we used a resolution of 0.25 for downstream analyses. The resulting Louvain clusters were visualized in a two-dimensional UMAP representation and were manually annotated with known cell types using canonical neural and non-neural marker genes, as well as signatures chosen in the original publications. We first distinguished neuronal cells from non-neuronal cells using the known pan-neural genes (*Snap25, Syp, Elval2, Slc17a6*, and *Slc32a1*) and canonical non-neural genes (*Olig1, Cldn5, C1qa, Sox9*, and *Mustn1*) (Fig. 3a,b).

### Characterization of cell subpopulations

To identify the various populations of excitatory and inhibitory neurons, we sub-setted the neuronal cell cluster of the integrated dataset and performed a second round of dimensional reduction and clustering using the FindVariableFeatures(), ScaleData(), RunPCA(), and RunUMAP() functions of the Seurat package. To group the cells with similar transcriptional profiles, we applied the resolution of the FindClusters() function as 0.3. We next examined the expression patterns of markers for excitatory and inhibitory neurons, which are *Slc17a6* for glutamatergic neurons and *Slc32a1*, *Gad1*, and *Gad2* for GABAergic neurons, respectively^35^. Of the 13 identified neuronal clusters, there are 4 glutamatergic clusters, 8 GABAergic clusters, and one cluster neither glutamatergic nor GABAergic, but *Cck* + (Supplementary Fig. S4a,b). Overall, within the identified neuronal cell populations, clusters can be identified by the unique combination of genes that are expressed at different levels. Most neuronal clusters contain potential sub-type markers and others are defined by combinatorial expression of marker genes. For example, GABA7 is specified with the high expression of arginine vasopressin (*Avp*), a neuropeptide controlling osmoregulation, and low expression of pro-opiomelanocortin (*Pomc*) neurons. Similarly, a neuropeptide such as *Agrp* that controls feeding, is highly expressed in the GABA6 cluster. Similarly, *Slc1a3, Nr4a2, Lhx1, Adcyap1, Lhx8, Crabp1*, and *Npy2r* are each expressed in only one neuron subtype, while *Htr2c, Six3, Meis2, Tubb3*, and *Tmem163* are each expressed in two or four neurons’ subtypes with distinct patterns (Supplementary Fig. S4c).

Next, we identified a total of 17 distinct clusters of non-neural cells. Oligodendrocytes were found using markers, such as *Olig1/2, Pdgfra, Fyn*, and *Mobp*, all of which show a gradient of gene expression in postnatal development^24,31,36,37^ (Supplementary Fig. S4d,e). These cells also include oligodendrocytes progenitor cells (OPC), newly formed oligodendrocytes (NFO), and myelinating oligodendrocytes (MO) that indicate axon myelination is still taking in the adult hypothalamus. We also identified one cluster as intermediate progenitor cells (IPCs) by the expression of *Hmgb2*, which encodes a chromatin-related transcription activator^38^. OPCs are another type of neural progenitor cells that express *Pdgfra*, which regulates processes of cell proliferation, differentiation, and migration through the Wnt and Notch signaling pathways. Subsequently, we also identified three distinct subtypes of astrocytes (Astro1, 2, and 3) based on overall levels of relative gene expression from none or low to high (Supplementary Fig. S4e,f). Each of these astrocyte subtypes expresses *Agt*, which regulates synaptic communication and cerebral blood flow at varying levels. The Astro1 cell cluster exhibits an elevated expression of proteins like *Gfap*, pivotal for cytoskeletal function, while the Astro2 cell cluster has a high expression of *C1qa* and a low expression of *Unc13c*^39,40^. The cells of Astro3 highly express *Unc13c* and *Rgcc*, which encode for membrane fusion and cell cycle regulation^41^. Tanycytes (Tany) and tanycyte-like ependymal cells (Tany-like ependy) were also found using the expression levels of *Rax* and *Ccdc153*, respectively, that have role in cell development and response to injury^24^. In addition, we found other distinct clusters readily identifiable as macrophages (Mac: *Mrc1*^+^), microglia (Micro: *Cx3cr1*^+^), mural cells (Mustn1^+^), two populations of endothelial cells (Endo1/2: *Pecam1*^+^), vascular leptomeningeal cells or VLMCs (*Dcn*^+^) and one erythrocyte representing cluster (Eryth: *Alas2*^+^). These findings of major non-neural cells obtained from the hypothalamus at varying postnatal stages closely align with previous scRNA-seq analyses from mouse brains^8,31,42,43^. Similarly, myelinating oligodendrocytes and tanycytes were subsetted to run a second round of dimensional reduction and clustering and thus to characterize cellular heterogeneity, (Supplementary Fig. S4g,h)^44–46^. Subtyping of myelinating oligodendrocytes (MO) revealed 3 clusters: (1) early mature oligodendrocytes (EMOL) expressing *Egr2* (Early Growth Response 2; also known as *Krox20*), during oligodendrocytes maturation, (2) mature oligodendrocytes (MOL) expressing late oligodendrocyte differentiation genes, such as *Klk6* and *Anxa5*, and (3) myelin-forming oligodendrocytes (Myl-OL) expressing genes involved in myelin formation (Supplementary Fig. S4g). Similarly, tanycytes were subdivided into four subtypes – α1, α2, β1, and β2 – based on their gene expression. Interestingly, we found one cell cluster expressing *S100b*, which is known to be involved in the regulation and migration of astrocytes^47^ (Supplementary Fig. S4h). This rigorous analysis yielded six additional sub-clusters, bringing the total number of non-neuronal subtypes to 23, further emphasizing the robust cellular differentiation of our integrated dataset. Finally, our large-scale integrated dataset surpasses the scope of individual studies by encompassing a broader array of a total of 36 distinct cell types that encompass all major and sub-cell types found in the hypothalamic regions, including glial-like cells such as ependymal cells, tanycytes, astrocytes, oligodendrocytes, previously undefined IPCs and neuronal cells with their sub-populations, thereby providing unprecedented perspective and insight into the hypothalamic molecular landscape. Furthermore, insight information on each cluster’s characteristics is detailed in Supplementary Table 2.

### Validation of transcriptional profiles

In our study, we utilized the integrated transcriptomic dataset as an established benchmarked reference to ascertain the accuracy of our nuConnect-seq dataset. The manually annotated cell types of the nuConnect-seq dataset were compared with the cell types of the integrated dataset using the ‘label transfer’ method from Seurat via the FindTransferAnchors() and TransferData() functions (Fig. 4a). These comparative approaches demonstrate that the projected cell types in our nuConnect-seq dataset closely match the cell types of the integrated reference dataset, validating the technical validity of the generated nuConnect-seq dataset (Fig. 4b). The histogram, merged UMAP and feature plots displaying the strength of the cell type predictions are shown in Fig. 4c,d, Supplementary Fig. S5 confirms the similarity of transcriptional profiles between neuronal and non-neuronal cell types in the two datasets. This transcriptional profiling comparison with an integrated transcriptomic reference dataset highlights the reliability and accuracy of our nuConnect-seq dataset.

### Supplementary information

Supplementary Table 1

Supplementary Table 2

Supplementary figures

## Data Availability

The R script used to generate the integrated dataset, conduct validation dataset analysis, and produce relevant plots can be found at: https://github.com/Junaid13913/PostnatalMouseHypothalamus_SingleCellTranscriptome. Our final datasets with UMAP embeddings are available at the figshare repository: (10.6084/m9.figshare.21981251.v1)^23^.
