# Supplementary material for: Unveiling Hypothalamic Molecular Signatures via Retrograde Viral Tracing and Single-Cell Transcriptomics
Source: Sci Data. 2023 Dec 4;10:861. doi: 10.1038/s41597-023-02789-6 (PMC10696032; doi:10.1038/s41597-023-02789-6)
Supplement: Supplementary file 3 — Supplementary figures [file 41597_2023_2789_MOESM3_ESM.pdf]

Table of contents for each supplementary figure

**Title**	**Page No.**
Supplementary Figure. 1	2
Supplementary Figure. 2	3
Supplementary Figure. 3	4
Supplementary Figure. 4	5
Supplementary Figure. 5	6

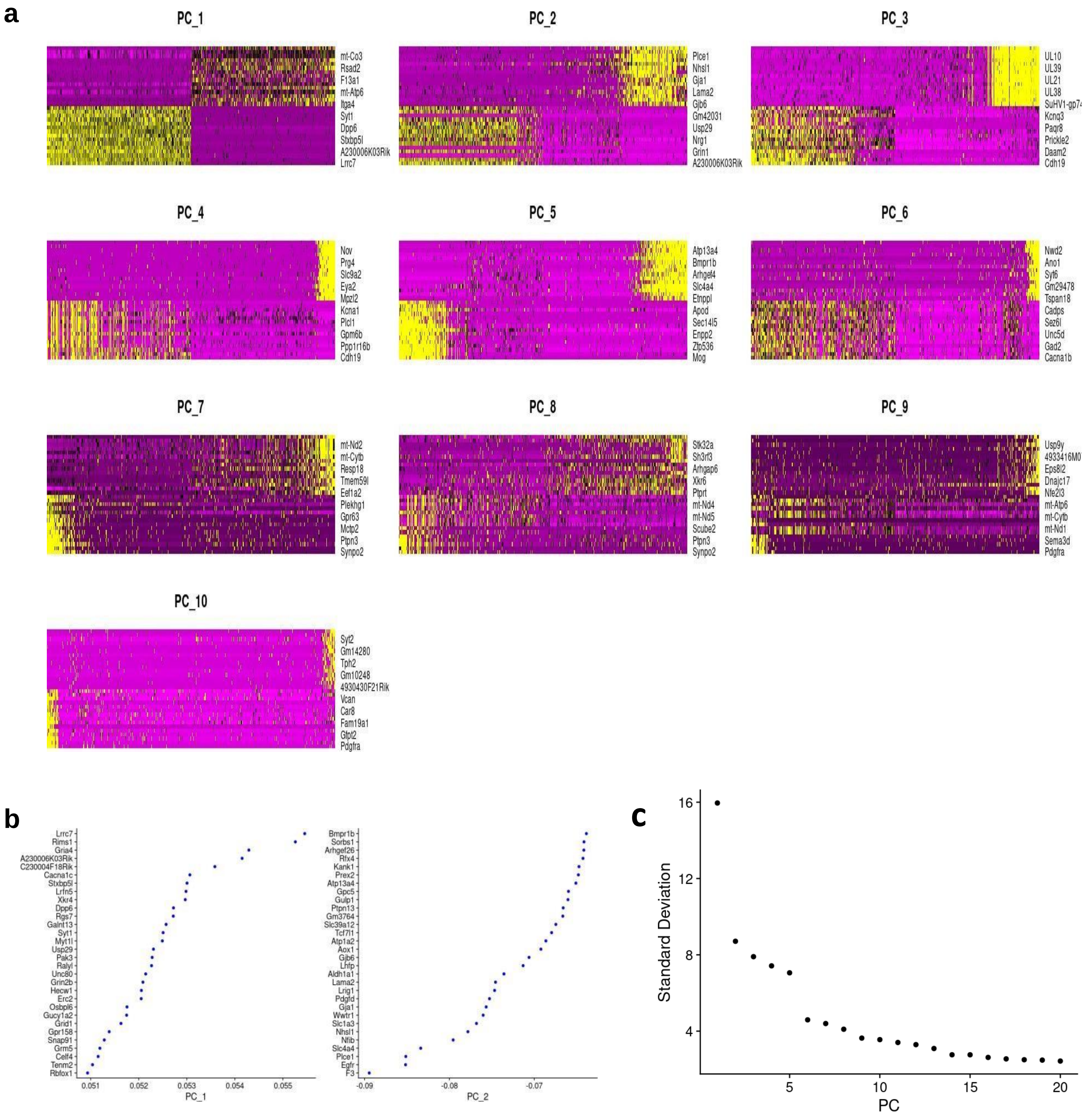

Figure S1. QC and processing of the nuConnect-seq dataset. (**a**, **b**) Heatmap and VizDimReduction plots show the distribution and ordering of both cells and features according to their PCA scores. (**c**) Elbowplot shows the ranking of principal components based on the percentage of variance explained by each one.

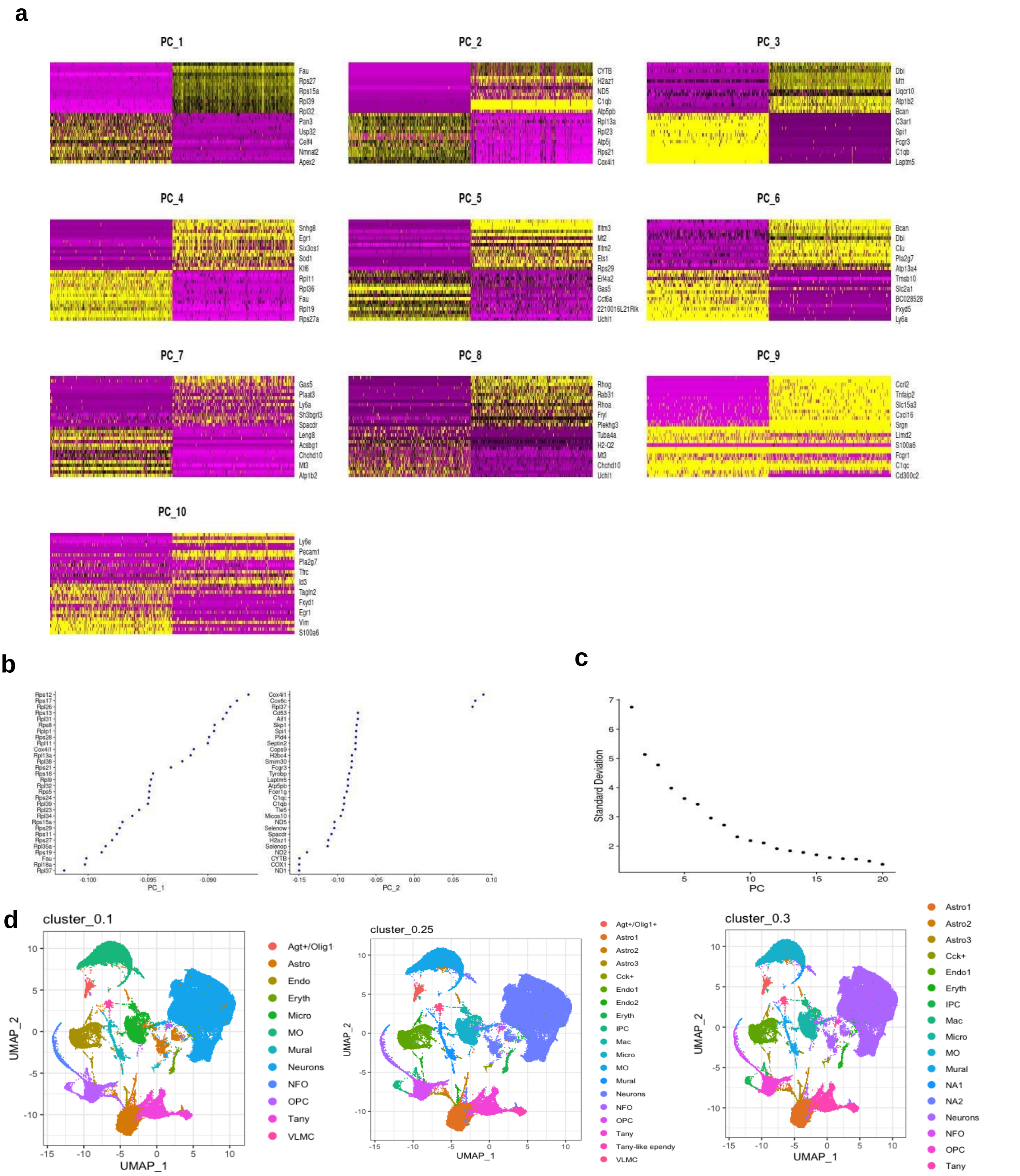

Figure S2. QC and processing of the integrated data. (**a**, **b**) Heatmap and VizDimReduction plots show the distribution and ordering of both cells and features according to their PCA scores. (**c**) Elbowplot shows the ranking of principle components based on the percentage of variance explained by each one. (**d**) UMAPs at various resolutions showing the granularity of clusters

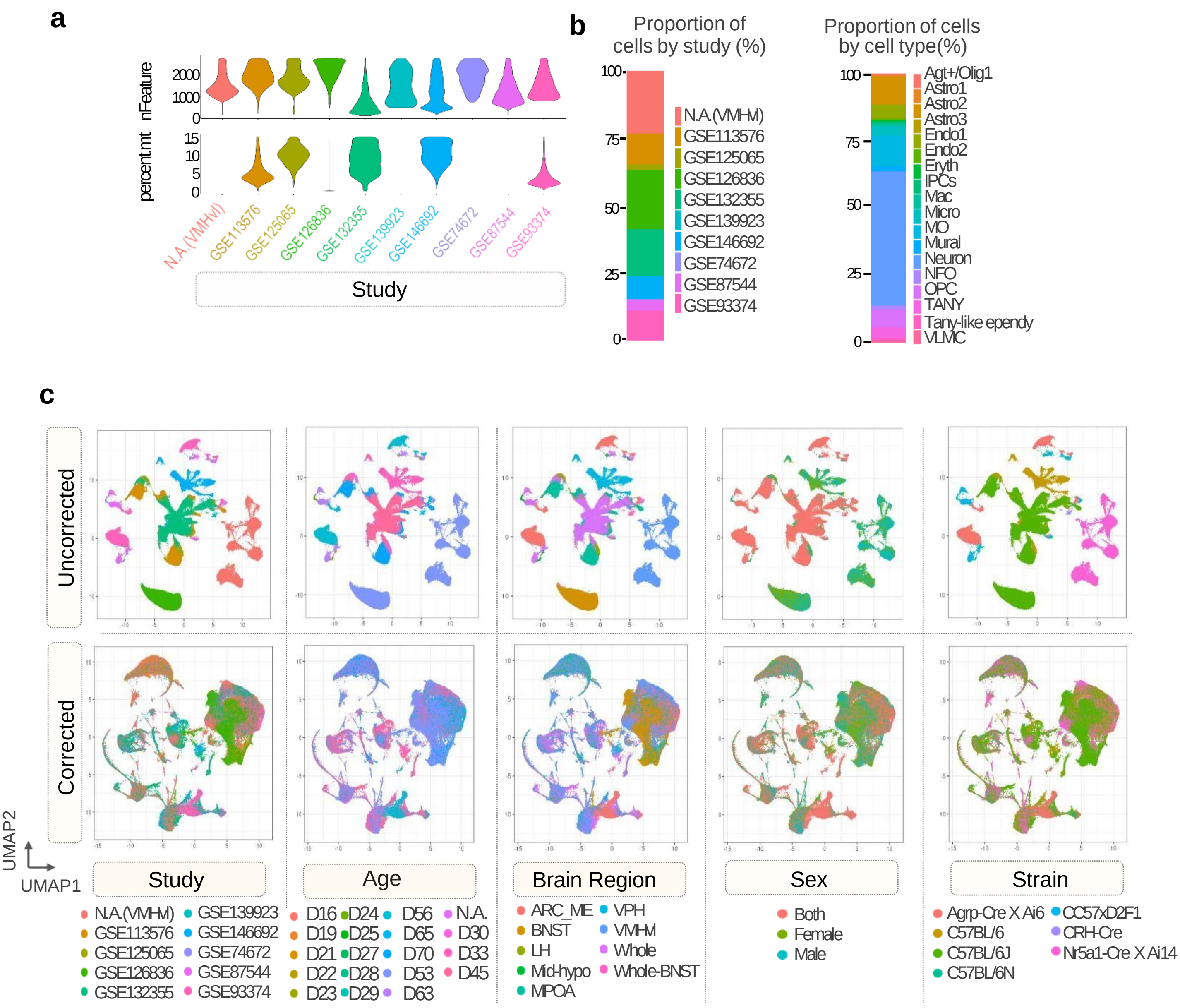

Figure S3. Cell type proportion and batch removal of the final integrated dataset. (**a**) Cells of the final integrated dataset are grouped by cell types, including the percentage of read counts from mitochondrial genes (percent. mt), and the number of unique genes (nFeature). (**b**) Proportions of cell numbers are grouped by study (left) and cell type (right),. (**c**) UMAP plots of the batch uncorrected (top) and corrected (bottom) integrated dataset. Cells are grouped by study, age, brain region, sex, and mouse strain.

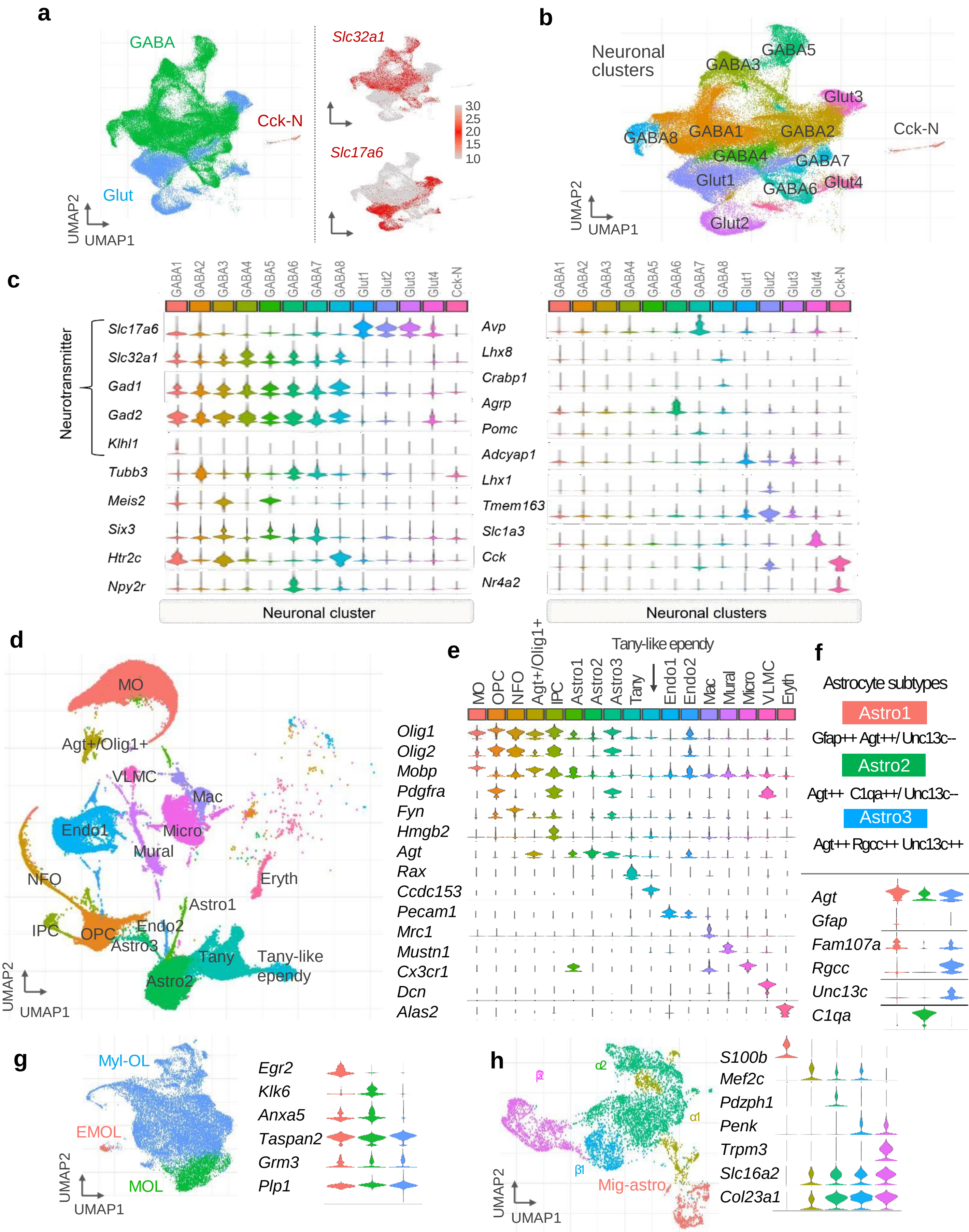

Figure S4. Cell types and sub-cell types of neuronal and non-neuronal clusters of the integrated dataset. (**a**) UMAP plots showing normalized expression of *Slc17a6*, and *Slc32a1* after the second iteration of unsupervised clustering on just neuronal cells. (**b**) UMAP plot showing 13 neuronal cell types identified by unsupervised clustering. (**c**) Relative expression of neurotransmitters (*Slc17a6*, *Slc32a1*, *Gad1*, *Gad2*) and discriminatory marker genes. (**d**) UMAP plot showing 17 non-neuronal cell types identified by unsupervised clustering. (**e**) Relative expression of cell type-specific genes differentially expressed across the 17 non-neuronal cell subtypes. (**f**) Relative expression of astrocyte subtype-specific genes. Positive and negative markers for each subtype are indicated by "++" and "--", respectively. (**g, h**) UMAP and violin plots show subtypes iteration of MO and tanycytes clusters.

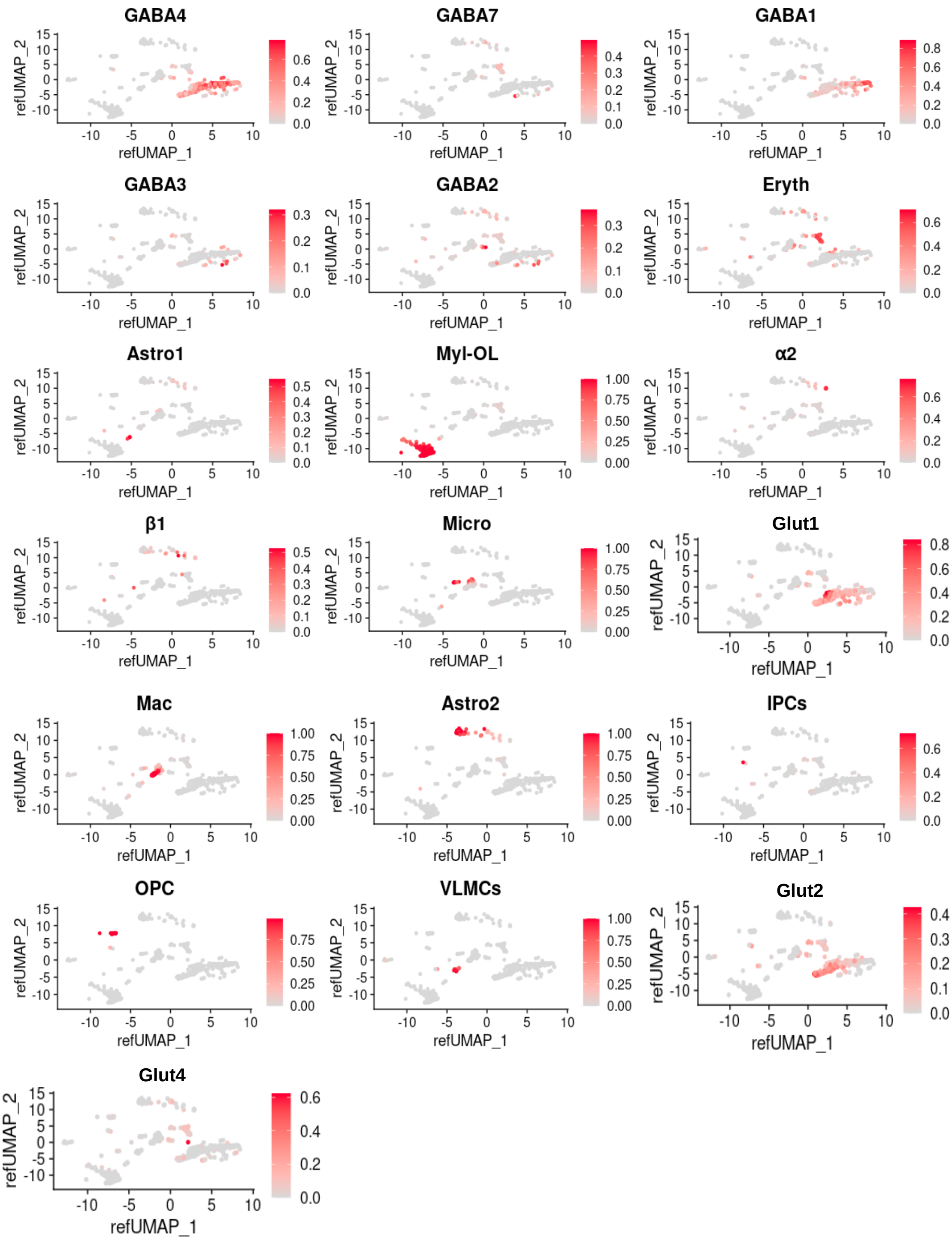

Figure S5. Feature plots showing predicted cell type on nuConnect-seq dataset after projecting cells onto integrated UMAPs.
